# 3-Cyano­anilinium hydrogen oxalate hemihydrate

**DOI:** 10.1107/S1600536812019824

**Published:** 2012-05-12

**Authors:** Xin-Yuan Chen

**Affiliations:** a Ordered Matter Science Research Center, College of Chemistry and Chemical Engineering, Southeast University, Nanjing 210096, People’s Republic of China

## Abstract

In the title hydrated mol­ecular salt, C_7_H_7_N_2_^+^·C_2_HO_4_^−^·0.5H_2_O, contains a 3-cyano­anilinium cation, a hydrogen oxalate anion and half a water mol­ecule in an asymmetric unit. The dihedral angle between the CO_2_(H) and CO_2_ planes of the hydrogen oxalate ion is 7.96 (1)°. In the crystal, the components are linked by N—H⋯O and O—H⋯O hydrogen bonds, forming a layer lying parallel to the *ac* plane.

## Related literature

For the properties of related compounds, see: Chen *et al.* (2000 ▶); Liu *et al.* (1999 ▶); Zhao *et al.* (2003 ▶). For the structures of related compounds, see: Dai & Chen (2011 ▶); Xu *et al.* (2011 ▶); Zheng (2011 ▶).
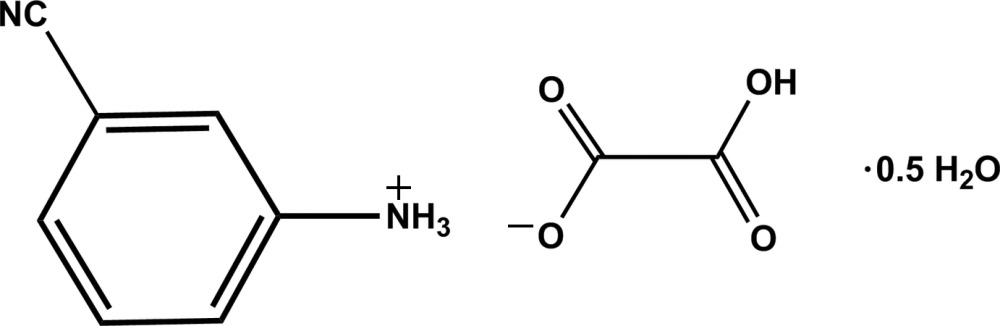


## Experimental

### 

#### Crystal data

C_7_H_7_N_2_^+^·C_2_HO_4_^−^·0.5H_2_O
*M* *_r_* = 217.18
Monoclinic, 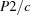

*a* = 15.1221 (7) Å
*b* = 5.6518 (1) Å
*c* = 13.6926 (6) Å
β = 113.22 (4)°
*V* = 1075.5 (3) Å^3^
*Z* = 4
Mo *K*α radiation
μ = 0.11 mm^−1^
*T* = 173 K
0.10 × 0.05 × 0.05 mm

#### Data collection

Rigaku Mercury2 (2 × 2 bin mode) diffractometer
Absorption correction: multi-scan (*CrystalClear*; Rigaku, 2005 ▶) *T*_min_ = 0.910, *T*_max_ = 1.000
7209 measured reflections
2446 independent reflections
1906 reflections with *I* > 2σ(*I*)
*R*_int_ = 0.034

#### Refinement

*R*[*F* ^2^ > 2σ(*F* ^2^)] = 0.054
*wR*(*F* ^2^) = 0.156
*S* = 1.07
2446 reflections
142 parameters
5 restraints
H-atom parameters constrained
Δρ_max_ = 0.28 e Å^−3^
Δρ_min_ = −0.24 e Å^−3^

### 

Data collection: *CrystalClear* (Rigaku, 2005 ▶); cell refinement: *CrystalClear*; data reduction: *CrystalClear*; program(s) used to solve structure: *SHELXS97* (Sheldrick, 2008 ▶); program(s) used to refine structure: *SHELXL97* (Sheldrick, 2008 ▶); molecular graphics: *SHELXTL* (Sheldrick, 2008 ▶); software used to prepare material for publication: *SHELXTL*.

## Supplementary Material

Crystal structure: contains datablock(s) I, global. DOI: 10.1107/S1600536812019824/pv2542sup1.cif

Structure factors: contains datablock(s) I. DOI: 10.1107/S1600536812019824/pv2542Isup2.hkl

Supplementary material file. DOI: 10.1107/S1600536812019824/pv2542Isup3.cml

Additional supplementary materials:  crystallographic information; 3D view; checkCIF report

## Figures and Tables

**Table 1:** Hydrogen-bond geometry (Å, °)

*D*—H⋯*A*	*D*—H	H⋯*A*	*D*⋯*A*	*D*—H⋯*A*
O1*W*—H1*WA*⋯O1^i^	0.82	1.96	2.767 (2)	166
N1—H1*A*⋯O1^ii^	0.89	1.91	2.797 (2)	172
N1—H1*C*⋯O2^iii^	0.89	1.96	2.778 (2)	152
O3—H3⋯O2^iii^	0.82	1.74	2.559 (2)	178
N1—H1*B*⋯O1*W*	0.89	1.91	2.788 (2)	167

Symmetry codes: (i) 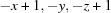; (ii) 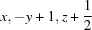; (iii) 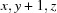.

**Fig. 1.**
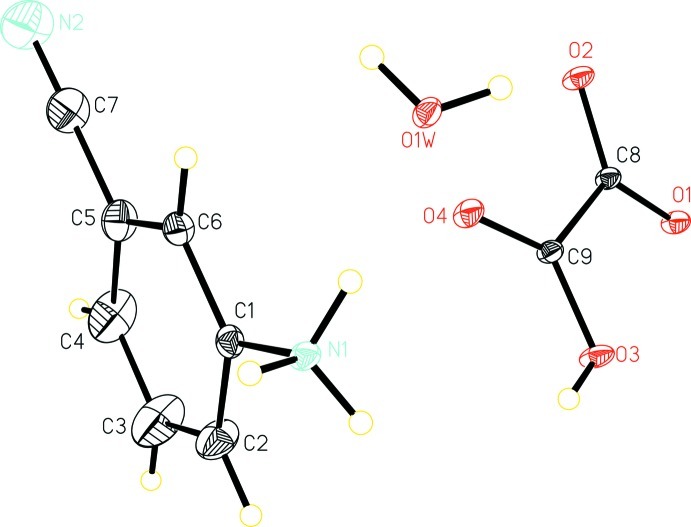
A view of the title compound with the atomic numbering scheme. Displacement ellipsoids were drawn at the 30% probability level.

**Fig. 2.**
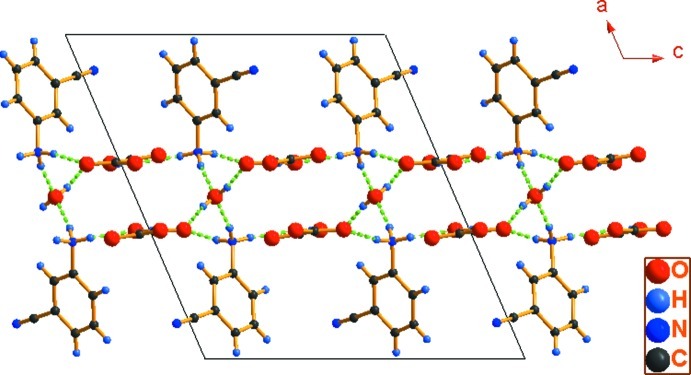
The crystal packing of the title compound viewing along the b-axis, showing the two-dimensional hydrogen-bonded network.

## supplementary crystallographic information

### Comment

Salts of amide attracted more attention as phase transition dielectric materials for its application in micro-electronics, memory storage (Chen *et al.*, 2000; Liu, *et al.* 1999; Zhao, *et al.* 2003). With the purpose of obtaining phase transition crystals of 3-aminobenzonitrile salts, its interaction with various acids has been studied and we have elaborated a serie of new materials with this organic molecule (Dai & Chen 2011; Xu, *et al.* 2011; Zheng 2011). In this paper, we describe the crystal structure of the title compound.

The asymmetric unit is composed of a 3-cyanoanilinium cation, a carboxyformate anion, and a half molecule of water (Fig. 1). The geometric parameters of the title compound agree well with reported similar structure (Dai & Chen 2011). The cation is almost planar (r.m.s. deviation 0.0062 Å, benzene ring as the best plane).

The cations are surrounded by the anions and water molecules *via* hydrogen bonds which play an important role in stabilizing the crystal structure. In the crystal structure, all the amino H atoms are involved in N—H···O hydrogen bonds with carboxyformate anion and water molecule with the distances of 2.797 (2) Å, 2.778 (2) Å and 2.788 (2) Å, respectively. In addition, the H atoms of water molecule and carboxyformate anion are involved in the O—H···O H-bonding interactions. In the crystal structure, those H-bonds link the ionic units into a two-dimensional sheets parallel to the *ac* plane (Table 1 and Fig. 2).

### Experimental

The commercial 3-aminobenzonitrile (3 mmol, 324 mg) and oxalic acid (3 mmol, 270 mg) were dissolved in 50 ml water/MeOH solution (1:1 *v*/*v*). The solvent was slowly evaporated in air affording colourless block-shaped crystals of the title compound suitable for X-ray analysis.

### Refinement

The H atoms were included in the refinement at geometrically idealized positions and treated in riding mode with O—H = 0.82 Å, N—H = 0.89 Å and C–H = 0.93 Å, with *U*_iso_(H) = 1.2*U*_e_q(C) or 1.5*U*_e_q(O/N); a rotating-group model was used for the –NH_3_ group.

### Crystal data

**Table:**

C_7_H_7_N_2_^+^·C_2_HO_4_^−^·0.5H_2_O	*F*(000) = 452
*M**_r_* = 217.18	*D*_x_ = 1.341 Mg m^−^^3^
Monoclinic, *P*2/*c*	Mo *K*α radiation, λ = 0.71073 Å
Hall symbol: -P 2yc	Cell parameters from 2446 reflections
*a* = 15.1221 (7) Å	θ = 2.9–27.5°
*b* = 5.6518 (1) Å	µ = 0.11 mm^−^^1^
*c* = 13.6926 (6) Å	*T* = 173 K
β = 113.22 (4)°	Block, colorless
*V* = 1075.5 (3) Å^3^	0.10 × 0.05 × 0.05 mm
*Z* = 4	

### Data collection

**Table:**

Rigaku Mercury2 (2x2 bin mode) diffractometer	2446 independent reflections
Radiation source: fine-focus sealed tube	1906 reflections with *I* > 2σ(*I*)
Graphite monochromator	*R*_int_ = 0.034
Detector resolution: 13.6612 pixels mm^-1^	θ_max_ = 27.5°, θ_min_ = 2.9°
CCD profile fitting scans	*h* = −18→19
Absorption correction: multi-scan (*CrystalClear*; Rigaku, 2005)	*k* = −7→7
*T*_min_ = 0.910, *T*_max_ = 1.000	*l* = −17→17
7209 measured reflections	

### Refinement

**Table:**

Refinement on *F*^2^	Primary atom site location: structure-invariant direct methods
Least-squares matrix: full	Secondary atom site location: difference Fourier map
*R*[*F*^2^ > 2σ(*F*^2^)] = 0.054	Hydrogen site location: inferred from neighbouring sites
*wR*(*F*^2^) = 0.156	H-atom parameters constrained
*S* = 1.07	*w* = 1/[σ^2^(*F*_o_^2^) + (0.0872*P*)^2^ + 0.1364*P*] where *P* = (*F*_o_^2^ + 2*F*_c_^2^)/3
2446 reflections	(Δ/σ)_max_ < 0.001
142 parameters	Δρ_max_ = 0.28 e Å^−^^3^
5 restraints	Δρ_min_ = −0.24 e Å^−^^3^

### Special details

**Table:**

Geometry. All e.s.d.'s (except the e.s.d. in the dihedral angle between two l.s. planes) are estimated using the full covariance matrix. The cell e.s.d.'s are taken into account individually in the estimation of e.s.d.'s in distances, angles and torsion angles; correlations between e.s.d.'s in cell parameters are only used when they are defined by crystal symmetry. An approximate (isotropic) treatment of cell e.s.d.'s is used for estimating e.s.d.'s involving l.s. planes.
Refinement. Refinement of *F*^2^ against ALL reflections. The weighted *R*-factor *wR* and goodness of fit *S* are based on *F*^2^, conventional *R*-factors *R* are based on *F*, with *F* set to zero for negative *F*^2^. The threshold expression of *F*^2^ > σ(*F*^2^) is used only for calculating *R*-factors(gt) *etc*. and is not relevant to the choice of reflections for refinement. *R*-factors based on *F*^2^ are statistically about twice as large as those based on *F*, and *R*- factors based on ALL data will be even larger.

### Fractional atomic coordinates and isotropic or equivalent isotropic displacement parameters (Å^2^)

**Table:**

	*x*	*y*	*z*	*U*_iso_*/*U*_eq_	
O1W	0.5000	0.3943 (3)	0.7500	0.0237 (4)	
H1WA	0.4708	0.3063	0.6999	0.036*	
N1	0.63809 (11)	0.7280 (2)	0.75668 (11)	0.0247 (4)	
H1A	0.6328	0.8305	0.8034	0.037*	
H1B	0.5962	0.6106	0.7469	0.037*	
H1C	0.6258	0.8012	0.6952	0.037*	
C1	0.73556 (13)	0.6324 (3)	0.79701 (14)	0.0278 (4)	
C6	0.75806 (14)	0.4443 (3)	0.86683 (15)	0.0312 (4)	
H6A	0.7120	0.3818	0.8888	0.037*	
C5	0.85041 (16)	0.3490 (4)	0.90408 (18)	0.0449 (6)	
C7	0.87338 (18)	0.1547 (5)	0.9797 (2)	0.0579 (7)	
C3	0.8941 (2)	0.6365 (8)	0.8017 (3)	0.0843 (11)	
H3A	0.9400	0.7024	0.7805	0.101*	
C2	0.80292 (17)	0.7312 (5)	0.7645 (2)	0.0547 (7)	
H2A	0.7870	0.8596	0.7183	0.066*	
N2	0.89001 (19)	0.0051 (5)	1.0408 (2)	0.0826 (9)	
C4	0.9188 (2)	0.4440 (7)	0.8704 (2)	0.0730 (9)	
H4A	0.9802	0.3793	0.8937	0.088*	
O1	0.60131 (9)	−0.0466 (2)	0.39238 (9)	0.0266 (3)	
O2	0.60400 (10)	−0.1979 (2)	0.54425 (9)	0.0294 (3)	
O3	0.60355 (10)	0.3895 (2)	0.46709 (9)	0.0302 (3)	
H3	0.6034	0.5206	0.4927	0.045*	
O4	0.62804 (10)	0.2420 (2)	0.62874 (9)	0.0298 (3)	
C9	0.61454 (12)	0.2186 (3)	0.53599 (13)	0.0216 (4)	
C8	0.60611 (12)	−0.0294 (3)	0.48550 (13)	0.0210 (4)	

### Atomic displacement parameters (Å^2^)

**Table:**

	*U*^11^	*U*^22^	*U*^33^	*U*^12^	*U*^13^	*U*^23^
O1W	0.0349 (10)	0.0193 (8)	0.0149 (8)	0.000	0.0076 (7)	0.000
N1	0.0353 (9)	0.0215 (7)	0.0201 (7)	−0.0007 (6)	0.0140 (6)	−0.0001 (6)
C1	0.0285 (10)	0.0333 (10)	0.0223 (8)	−0.0041 (7)	0.0107 (7)	−0.0045 (7)
C6	0.0315 (10)	0.0308 (10)	0.0305 (10)	−0.0006 (7)	0.0115 (8)	−0.0024 (8)
C5	0.0347 (12)	0.0507 (14)	0.0439 (13)	0.0079 (10)	0.0097 (9)	0.0010 (10)
C7	0.0420 (14)	0.0564 (16)	0.0646 (17)	0.0147 (11)	0.0097 (12)	0.0095 (14)
C3	0.0396 (16)	0.146 (3)	0.076 (2)	0.0062 (17)	0.0328 (15)	0.039 (2)
C2	0.0386 (13)	0.0800 (19)	0.0483 (14)	−0.0052 (11)	0.0200 (11)	0.0215 (13)
N2	0.0643 (16)	0.0727 (18)	0.092 (2)	0.0214 (13)	0.0107 (14)	0.0307 (15)
C4	0.0335 (14)	0.118 (3)	0.0714 (19)	0.0210 (15)	0.0244 (13)	0.0192 (19)
O1	0.0457 (8)	0.0193 (6)	0.0186 (6)	−0.0043 (5)	0.0168 (5)	−0.0029 (5)
O2	0.0541 (9)	0.0158 (6)	0.0209 (6)	−0.0013 (5)	0.0177 (6)	0.0005 (5)
O3	0.0592 (9)	0.0138 (6)	0.0219 (6)	−0.0003 (5)	0.0205 (6)	0.0005 (5)
O4	0.0505 (9)	0.0228 (7)	0.0177 (6)	−0.0020 (5)	0.0154 (5)	−0.0032 (5)
C9	0.0320 (9)	0.0166 (8)	0.0170 (8)	−0.0012 (6)	0.0105 (7)	0.0001 (6)
C8	0.0304 (9)	0.0158 (8)	0.0176 (8)	−0.0006 (6)	0.0104 (6)	0.0000 (6)

### Geometric parameters (Å, º)

**Table:**

O1W—H1WA	0.8207	C3—C2	1.376 (4)
N1—C1	1.459 (2)	C3—C4	1.389 (5)
N1—H1A	0.8900	C3—H3A	0.9300
N1—H1B	0.8900	C2—H2A	0.9300
N1—H1C	0.8900	C4—H4A	0.9300
C1—C6	1.380 (3)	O1—C8	1.2521 (19)
C1—C2	1.380 (3)	O2—C8	1.255 (2)
C6—C5	1.392 (3)	O3—C9	1.314 (2)
C6—H6A	0.9300	O3—H3	0.8205
C5—C4	1.395 (4)	O4—C9	1.211 (2)
C5—C7	1.454 (4)	C9—C8	1.546 (2)
C7—N2	1.146 (4)		
			
C1—N1—H1A	109.5	C2—C3—C4	121.2 (3)
C1—N1—H1B	109.5	C2—C3—H3A	119.4
H1A—N1—H1B	109.5	C4—C3—H3A	119.4
C1—N1—H1C	109.5	C3—C2—C1	118.8 (2)
H1A—N1—H1C	109.5	C3—C2—H2A	120.6
H1B—N1—H1C	109.5	C1—C2—H2A	120.6
C6—C1—C2	121.57 (19)	C3—C4—C5	119.3 (2)
C6—C1—N1	118.97 (16)	C3—C4—H4A	120.3
C2—C1—N1	119.46 (18)	C5—C4—H4A	120.3
C1—C6—C5	119.32 (19)	C9—O3—H3	112.2
C1—C6—H6A	120.3	O4—C9—O3	126.41 (15)
C5—C6—H6A	120.3	O4—C9—C8	121.21 (15)
C6—C5—C4	119.8 (2)	O3—C9—C8	112.36 (13)
C6—C5—C7	118.5 (2)	O1—C8—O2	125.99 (15)
C4—C5—C7	121.7 (2)	O1—C8—C9	119.18 (14)
N2—C7—C5	177.9 (3)	O2—C8—C9	114.83 (14)

### Hydrogen-bond geometry (Å, º)

**Table:**

*D*—H···*A*	*D*—H	H···*A*	*D*···*A*	*D*—H···*A*
O1*W*—H1*WA*···O1^i^	0.82	1.96	2.767 (2)	166
N1—H1*A*···O1^ii^	0.89	1.91	2.797 (2)	172
N1—H1*C*···O2^iii^	0.89	1.96	2.778 (2)	152
O3—H3···O2^iii^	0.82	1.74	2.559 (2)	178
N1—H1*B*···O1*W*	0.89	1.91	2.788 (2)	167

Symmetry codes: (i) −*x*+1, −*y*, −*z*+1; (ii) *x*, −*y*+1, *z*+1/2; (iii) *x*, *y*+1, *z*.
